# Path Loss Modeling for RIS-Assisted Wireless Communication in Tunnel Scenarios

**DOI:** 10.3390/s25041247

**Published:** 2025-02-18

**Authors:** Qi Yang, Yating Wu, Hengkai Zhao, Yichen Feng, Yanqiong Sun, Zhou Fang, Guoxin Zheng

**Affiliations:** 1 Key Laboratory of Specialty Fiber Optics and Optical Access Networks, Joint International Research Laboratory of Specialty Fiber Optics and Advanced Communication, Shanghai University, Shanghai 200444, China; oxygen7@shu.edu.cn (Q.Y.); fengyichen98@shu.edu.cn (Y.F.); fangzhou419@shu.edu.cn (Z.F.); gxzheng@staff.shu.edu.cn (G.Z.); 2 CASCO Signal Ltd., Shanghai 200072, China; sunyanqiong@casco.com.cn

**Keywords:** reconfigurable intelligent surface, millimeter wave, path loss, tunnel

## Abstract

Aiming to address the problem of limited transmission distance in applying reconfigurable intelligent surface (RIS) technology, this study leverages a tunnel simulation platform to investigate RIS-assisted wireless communication systems. Through theoretical derivation, we propose a path loss model formula specifically applicable to tunnel scenarios. Simulation results demonstrate that the proposed model accurately reflects the communication performance characteristics of RIS in tunnel scenarios, verifying the capability of RIS technology to enhance signal transmission distance within tunnels in rail transit engineering applications. This finding highlights the significant engineering potential and value of RIS technology.

## 1. Introduction

Millimeter wave (mmWave) wireless signals are well suited for basic line-of-sight (LOS) scenarios and low-penetration quasi-LOS environments. However, they face challenges in effectively covering outdoor non-line-of-sight (NLOS) scenarios where buildings obstruct the signal and indoor environments with high penetration loss [1,2].

The emergence of RIS technology presents a novel approach to addressing the challenges of transmitting high-frequency wireless signals, particularly in scenarios with severe propagation constraints, and has thus garnered significant academic attention. In the context of RIS-assisted wireless channel transmission, Lan et al. [3] investigated the channel characteristics of RIS at 26 GHz in outdoor scenarios using a vector network analyzer. Basar et al. [4] developed a precise, open-source, and widely applicable mmWave RIS channel model suitable for indoor and outdoor environments. This model also incorporates realistic channel characteristics for the fifth-generation mobile networks (5G) scenarios involving RIS, and they further introduced a comprehensive channel simulator for simulating RIS-assisted communication systems.

Extensive research has been conducted to develop path loss models that incorporate the distinct electromagnetic characteristics of near-field and far-field conditions. Tang et al. [5] formulated RIS free space path loss models for both near-field and far-field scenarios, elucidating the relationships between RIS path loss and key factors such as the transmitter-receiver distance, unit size, near-field/far-field effects, and antenna radiation patterns. These models were rigorously validated through simulations and experiments. Building on this work, Tang et al. [6] refined the framework to estimate better the path loss for RIS structures composed of sub-wavelength-sized units. El-Absi et al. [7] extended the analysis to radio-frequency identification (RFID) systems by introducing a path loss model that incorporates radar cross-section (RCS), RIS physical characteristics, and near-field/far-field effects. Experimental results confirmed the model’s strong alignment with actual channel measurements. Zhou et al. [8] proposed a dual-path propagation approach leveraging vertically positioned RIS, simulating variations in received power under different conditions. Furthermore, Wang et al. [9] developed an innovative received power model based on RCS, which accurately predicts received power in RIS-assisted communication systems and captures the influence of angular variations on the reflection phase.

The academic community has extensively explored the application of RIS technology in various scenarios, such as mobile edge computing, unmanned aerial vehicle (UAV) communications, and integrated sensing and communication (ISAC) [10,11,12,13,14,15]. However, research on the application of RIS in railway transportation scenarios remains relatively limited. For example, Zhang et al. [16] proposed a novel RIS-assisted high-speed railway wireless communication paradigm, addressing key challenges and identifying potential application scenarios. The study demonstrates that deploying RIS on walls, tracks, and window glass can enhance data rates by 19%, 24%, and 29%, respectively. Additionally, research in [17] presents a RIS-based interference suppression technique to improve communication quality in railway wireless systems by maximizing the signal-to-interference-plus-noise ratio (SINR).

Existing studies primarily focus on theoretical research on RIS but have yet to model the wireless channel propagation characteristics of RIS in tunnel scenarios. This paper presents a path loss model for RIS in tunnel scenarios by extending the general path loss formula to bridge this research gap. We validate the model using the Wireless InSite (WI) software, providing a theoretical foundation for the engineering application of RIS in tunnel scenarios. The main innovations of this paper are summarized as follows:

- Field measurements were conducted on Shanghai Metro Line 7, and the measurement data were used to calibrate the material parameters of the ray-tracing (RT) model.
- Based on tunnel propagation characteristics and incorporating RT methods and electromagnetic theory, we derived a RIS path loss model applicable to tunnel scenarios, providing a theoretical basis for the design and optimization of tunnel wireless communication systems.
- We constructed an RIS-assisted tunnel wireless communication simulation platform using WI software. Based on the calibrated parameters, we designed a simulation scenario, and the results verified the effectiveness of the proposed path loss model.
- To further improve the adaptability and reliability of the model, a t-distribution parameter based on the difference between theory and simulation is introduced so that the model can more comprehensively reflect the uncertainty in the actual communication environment.

The remaining structure of this paper is as follows: Section 2 explains the construction of the tunnel channel simulation platform based on RT principles. By comparing the measured and simulated path loss results, the correctness of the simulation platform is verified. Section 3 analyzes existing path loss formulas and derives a new path loss formula for RIS in tunnel scenarios. Section 4 constructs a simulation scenario for RIS-assisted wireless communications in tunnels and validates the proposed path loss formula through simulations. Finally, Section 5 summarizes the study.

## 2. Calibration of Simulation Parameters for Tunnel Scenarios

### 2.1. Measurement Platform and Simulation Setup for Tunnel Scenarios

**Figure 1:**
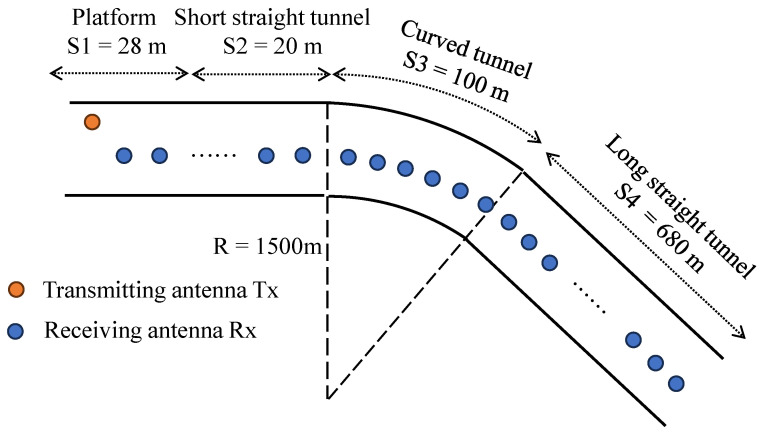
Shanghai Metro Line 7 layout.

The tunnel measurement was conducted between Shanghai University Station and Qihua Road Station on Shanghai Metro Line 7. The tunnel has a radius of 2.775 m, and its overall tunnel structure consists of four segments: platform, short straight tunnel, curved tunnel, and long straight tunnel, as illustrated in Figure 1. Considering the limited opportunities for conducting actual measurements in mmWave tunnels, it is essential to calibrate simulation parameters using data from measured scenarios. This calibration ensures the accuracy and reliability of subsequent simulation experiments, enabling the exploration of channel characteristics across a broader range of scenarios.

### 2.2. Simulation Calibration Based on Measured Data

Using Autodesk Inventor software, we constructed a three-dimensional (3D) simulation model based on the tunnel environment of Shanghai Metro Line 7. After importing the model into the WI, we calibrated and adjusted it to ensure accuracy and precision. The simulation parameters and antenna parameters were set according to the measurement environment. Table 1 provides the basic antenna configuration in the tunnel scenario.

**Figure 2:**
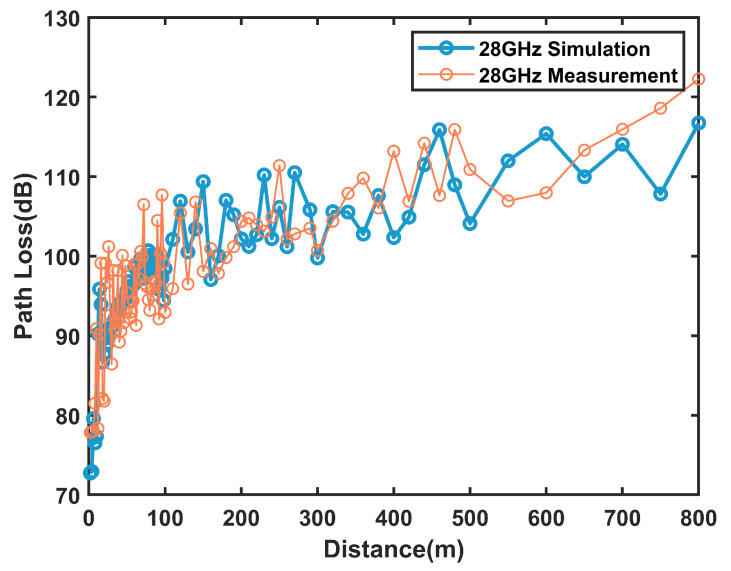
Comparison of path loss at 28 GHz in the tunnel scenario.

After adjusting the simulation parameters several times, the Mean Absolute Error (MAE), which is denoted as Equation (1), was calculated as 4.5 dB. The comparison results between measurement and simulation are shown in Figure 2, which shows that the error between measurement and simulation is small and that the fitted parameters are more credible.

(1) $$MAE=\frac{\sum_{i=1}^{N}\left|R_{cal}-R_{sim}\right|}{N}$$

where $R_{cal}$ denotes the data to be calibrated, $R_{sim}$ denotes the simulation data, and *N* denotes the number of receiving antennas (Rx).

The calibrated simulation parameters are presented in Table 2. As detailed in Section 4, these calibrated parameters will be used to construct simulation scenarios for RIS-assisted wireless communication performance in the tunnel scenario.

## 3. Wireless Channel Propagation Characteristics in Tunnel Scenarios

### 3.1. Classical Path Loss Models

The free space path loss model, which describes power attenuation during wireless signal propagation from the transmitting antenna (Tx) to the Rx, is based on the Friis transmission formula. Its expression is as follows [18]:

(2) PL(d)=32.45+20lg(f)+20lg(d)

where *d* is the distance between Tx and Rx in kilometers, and *f* is the frequency in MHz.

To accommodate more complex propagation environments, the classical log-distance path loss model extends the free space model by introducing the path loss exponent *n* and the shadow fading random variable $X_{\sigma }$. The path loss model is expressed as [18]:

(3) $$PL\left(d\right)=PL\left(d_{0}\right)+10n\mathrm{lg}\left(\frac{d}{d_{0}}\right)+X_{\sigma }\;$$

where $d_{0}$ is the reference distance, typically set to 1 m in tunnel scenarios. *n* denotes the path loss exponent and is equal to 2 in free space propagation scenarios. $X_{\sigma }$ is a random variable with a mean of zero and a standard deviation of σ, following a Gaussian distribution to characterize the randomness of shadowing effects.

The single-frequency floating intercept model is a simplified version of the log-distance model. It assumes that the path loss intercept $PL(d_{0})$ is not a fixed value but a floating parameter determined through data fitting. The model is expressed as:

(4) PL(d)=A+10nlg(d)

where *A* is the floating intercept, by removing the shadow fading term $X_{\sigma }$, the single-frequency floating intercept model focuses on the variation of path loss with distance, making it suitable for single-frequency scenarios or propagation environments with low sensitivity to shadowing effects.

**Figure 3:**
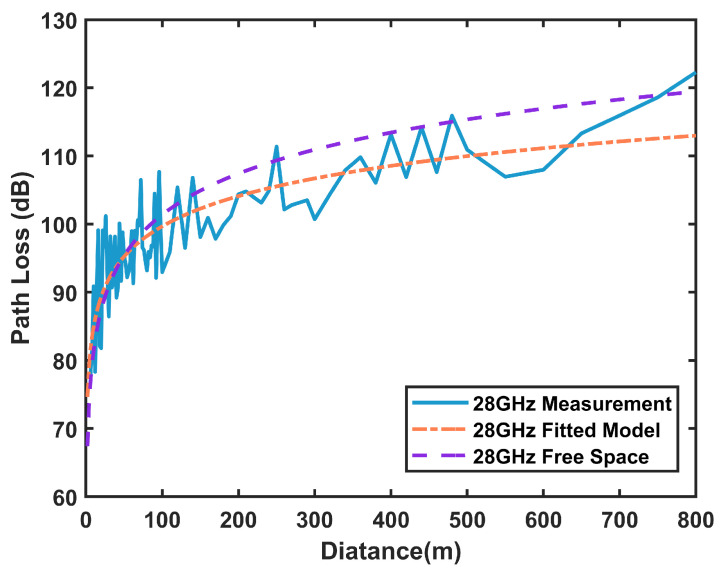
Comparison of path loss between tunnel and free space scenarios.

In summary, these three models have distinct characteristics and applicability. Figure 3 shows the measured path loss, the path loss in free space, and the path loss of the floating intercept model using Equation (4) in the subway tunnel scenario when the center frequency is 28 GHz.

As shown in Figure 3, the received power in the tunnel scenario is consistently lower than in the free space propagation. By substituting different frequency values into the above path loss model, the corresponding path loss can be calculated to assist in frequency selection. Additionally, the relationship between the transmitter-receiver distance and path loss provides a basis for the rational deployment of base station spacing.

### 3.2. Path Loss Modeling for RIS in Free Space

RIS enhances signal propagation by incorporating controllable reflecting elements into the wireless transmission paths, significantly reducing path loss. Refs. [5,6] proposed a general path loss model for RIS-assisted wireless communication, focusing on the free space path loss model of the reflection-assisted path provided by the intelligent metasurface, outlined in Equation (5), which considers various factors in RIS systems, including distance, frequency, antenna gain, and more.

(5) $$PL=\frac{P_{t}}{P_{r}}=\frac{16\pi^{2}}{G_{t}G_{r}{\left(d_{x}d_{y}\right)}^{2}{\left|\sum_{m=1-\frac{M}{2}}^{\frac{M}{2}}\sum_{n=1-\frac{N}{2}}^{\frac{N}{2}}\frac{\sqrt{F_{n,m}^{combine}}\Gamma_{n,m}}{r_{n,m}^{t}r_{n,m}^{r}}e^{\frac{-j2\pi \left(r_{n,m}^{t}+r_{n,m}^{r}\right)}{\lambda }}\right|}^{2}}$$

where $P_{t}$ represents the transmitted power, $P_{r}$ represents the received power, *N* and *M* represent the number of rows and columns of the unit, λ represents the signal wavelength, $G_{t}$ and $G_{r}$ represent the gains of the Tx and Rx, $d_{x}$ and $d_{y}$ represent the length and width of each RIS unit, $r_{n,m}^{t}$ and $r_{n,m}^{r}$ represent the distances from the Tx and Rx to the *n*th row and *m*th column unit, $\Gamma_{n,m}$ is the programmable reflection coefficient of the RIS reflective unit, and $F_{n,m}^{combine}$ represents the normalized power radiation pattern of the joint Tx, Rx, and RIS unit, which is related to the angle. As shown below:

(6) $$F_{n,m}^{combine}={\left(\cos\theta_{n,m}^{tx}\right)}^{\left(\frac{G_{t}}{2}-1\right)}{\left(\cos\theta_{n,m}^{t}\right)}^{\alpha }{\left(\cos\theta_{n,m}^{r}\right)}^{\alpha }{\left(\cos\theta_{n,m}^{rx}\right)}^{\left(\frac{G_{r}}{2}-1\right)}$$

where $\theta_{n,m}^{t}$ and $\theta_{n,m}^{r}$ represent the elevation angle from the RIS unit to the Tx and the elevation angle from the Rx, respectively, $\theta_{n,m}^{tx}$ and $\theta_{n,m}^{rx}$ represent the elevation angle from the Tx and the Rx to the RIS unit. The coefficient α is used to fit the actual radiation pattern of the electromagnetic element and is related to the specific design of the electromagnetic element.

**Figure 4:**
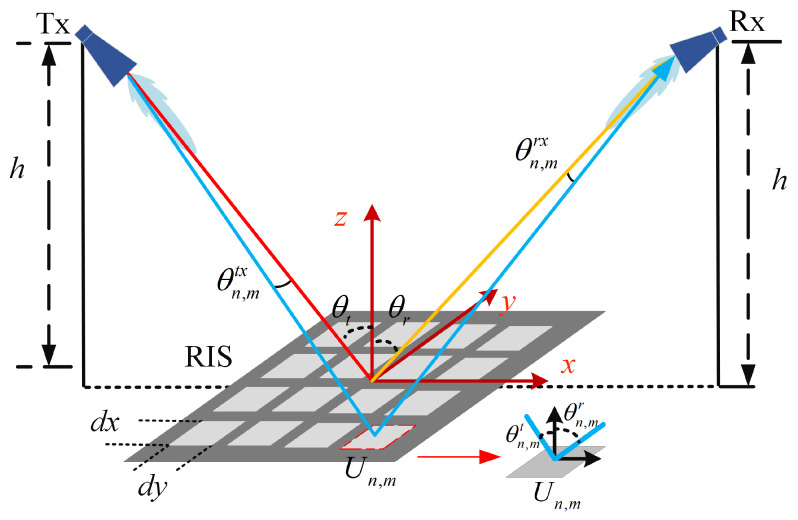
RIS-assisted wireless communication system.

**Figure 5:**
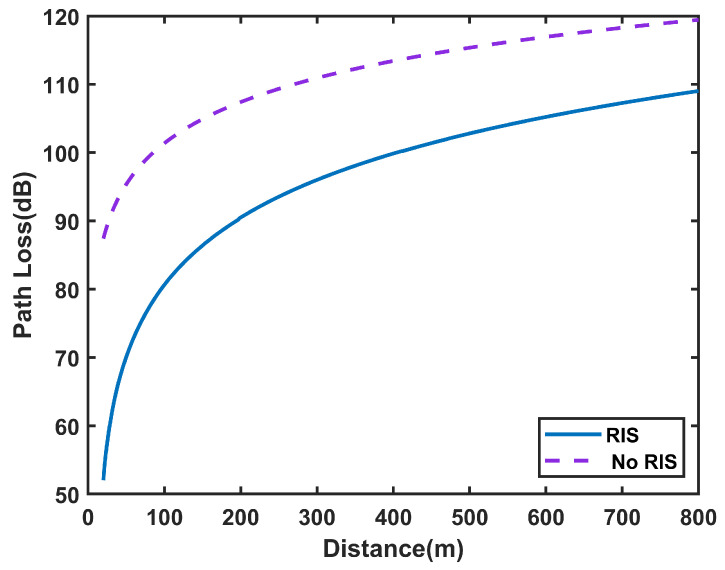
Path loss variation with distance for RIS in free space.

Based on the above studies, considering the RIS-assisted wireless communication system shown in Figure 4, we focus on the path loss characteristics of RIS in free space. Numerical simulations were conducted using Equation (5) to investigate the path loss variation with the distance between Tx and Rx. The simulation parameters are shown in Table 3. The distance between Tx and Rx was set to range from 20 m to 800 m to explore the trend of path loss when RIS is deployed. The simulation results are presented in Figure 5.

As shown in Figure 5, using RIS effectively reduces path loss in wireless communication, further verifying the potential of RIS to improve communication performance in free space.

### 3.3. Theoretical Path Loss Model for RIS in Tunnel Scenarios

Based on the ray tracing method and the theoretical foundation of the electromagnetic field, we propose a theoretical model of path loss based on RIS in tunnel scenarios, which combines RT algorithms with Geometric Optics (GO) and the Uniform Theory of Diffraction (UTD) [19,20,21]. In practical wireless environments, the received power is derived from the superposition of the electric field components from all arriving paths. Specifically, the electric field strength of each path incorporates parameters such as directional gain and relative phase. By integrating the effects of these physical quantities and electromagnetic parameters, the received power $P_{r}$ can be expressed as:

(7) $$P_{r}=\frac{\lambda^{2}\beta }{8\pi \eta_{0}}{\left|{\vec{E}}_{total}\right|}^{2}$$

where λ denotes the wavelength, $\eta_{0}$ represents the impedance of free space (approximately 377 Ω). β represents the spectrum overlap factor, which quantifies the degree of alignment between the spectrum of the receiver and the transmitter, thereby reflecting the frequency-matching condition. This factor is typically flatly distributed in narrowband systems and can be approximated as a constant value of 1.

In multipath propagation, the total electric field ${\vec{E}}_{total}$ at the receiving point is obtained by summing the electric field components from all propagation paths:

(8) $${\vec{E}}_{total}=\sum_{i=1}^{N_{p}}{\vec{E}}_{i}$$

where $N_{P}$ is the number of propagation paths between Tx and Rx.

The electric field component ${\vec{E}}_{i}$ of each path, weighted by the directional gain functions $g_{\theta }\left(\theta_{i},\phi_{i}\right)$ and $g_{\phi }\left(\theta_{i},\phi_{i}\right)$, can be expressed as:

(9) $${\vec{E}}_{i}=E_{\theta ,i}\left(\theta_{i},\phi_{i}\right)g_{\theta }\left(\theta_{i},\phi_{i}\right)+E_{\phi ,i}\left(\theta_{i},\phi_{i}\right)g_{\phi }\left(\theta_{i},\phi_{i}\right)$$

where $E_{\theta ,i}\left(\theta_{i},\phi_{i}\right)$ and $E_{\phi ,i}\left(\theta_{i},\phi_{i}\right)$ are the electric field amplitudes along the θ-direction and ϕ-direction for path *i*, respectively. $g_{\theta }\left(\theta_{i},\phi_{i}\right)$ and $g_{\phi }\left(\theta_{i},\phi_{i}\right)$ are the directional gain functions, representing the antenna gain for the electric field in the θ- and ϕ-directions, as described in Equation (10).

(10) $$g_{\theta }\left(\theta ,\phi \right)=\sqrt{G_{\theta }\left(\theta ,\phi \right)}e^{j\psi_{\theta }}$$

where $G_{\theta }\left(\theta ,\phi \right)$ represents the gain of the receiving antenna in the θ-direction, and $\psi_{\theta }$ denotes the relative phase, describing the phase change of the electric field. Similarly, $g_{\phi }\left(\theta ,\phi \right)$ is defined. Through this definition of the gain function, the complex electric field components of each path at the receiving point can be expressed, including both amplitude and phase information.

Considering the influence of the RIS, the electric field components consist of the field ${\vec{E}}_{direct}$, which is unaffected by RIS reflection or transmission, and the field ${\vec{E}}_{RIS}$, which is reflected by the RIS. The directional components of ${\vec{E}}_{direct}$ are further decomposed as shown in Equation (11).

(11) $$E_{\theta ,direct}\left(\theta ,\phi \right)=\sqrt{\frac{P_{t}\eta_{0}}{2\pi }}\sqrt{\left|G_{\theta }\left(\theta ,\phi \right)\right|}e^{j\psi_{\theta }}\frac{e^{-jkr_{TR}}}{r_{TD}}R_{G}^{\|}\prod_{n=1}^{N}R_{n}^{⊥}D_{s}\sqrt{\frac{r_{TD}}{r_{DR}\left(r_{TD}+r_{DR}\right)}}\prod_{m=1}^{M}R_{m}^{⊥}$$

where $R_{n}^{⊥}$ represents the reflection coefficient of the *n*-th reflection path perpendicular to the incident plane. Similarly, $R_{G}^{\|}$ denotes the ground reflection coefficient of the electric field component parallel to the incident plane. Additionally, $r_{TD}$ signifies the distance from the Tx to the point of circumference, while $r_{DR}$ represents the distance from the Rx to the aforementioned point. $D_{s}$ denotes the coefficient of circumference, $R_{m}^{⊥}$ is the reflection coefficient of the *M* reflection paths before diffraction. Notably, the electric field component in the ϕ-direction exhibits a similar pattern to that observed in the θ-direction.

When utilizing RIS, De et al. [22] demonstrated how metasurfaces can control the reflection, transmission, and scattering characteristics of electromagnetic waves under specific design conditions. The electric field reflected by the RIS, $E_{\mathrm{RIS}}$, is expressed as shown in Equation (12).

(12) $${\vec{E}}_{RIS}={\vec{E}}_{a}+\Gamma {\vec{E}}_{a}e^{jk\psi }+T{\vec{E}}_{a}e^{jk\psi }$$

where ${\vec{E}}_{a}$ is the incident electric field, Γ and T represent the reflected and transmitted electric field coefficients, respectively, *k* denotes the wave number, and ψ is the phase factor describing the metasurface.

Path loss is a quantity that represents signal strength attenuation in wireless propagation and is usually defined as the ratio of transmitted and received power in dB. We introduce the gains $G_{t}$ and $G_{r}$ of the Tx and Rx and consider the additional system loss $L_{s}$ to obtain the general definition of path loss as follows:

(13) $$PL=P_{t}\;-\;P_{r}\;+\;G_{r}\;+\;G_{t}\;-\;L_{s}$$

Substituting the expression for $P_{r}$ into Equation (13), the path loss expression for RIS-based communication in tunnel scenarios can be formulated as:

(14) $$PL_{1}=P_{t}-10\mathrm{lg}(\frac{\lambda^{2}\beta }{8\pi \eta_{0}}{\left|\sum_{i=1}^{N_{p}}\left[E_{\theta ,i}\left(\theta_{i},\phi_{i}\right)g_{\theta }\left(\theta_{i},\phi_{i}\right)+E_{\phi ,i}\left(\theta_{i},\phi_{i}\right)g_{\phi }\left(\theta_{i},\phi_{i}\right)\right]\right|}^{2})-30+G_{r}+G_{t}-L_{s}$$

where $L_{s}$ is the remaining loss of the communication system, the transmitted power is set as a known quantity $P_{t}$, $G_{t}$ and $G_{r}$ represent the gains of Tx and Rx.

## 4. Performance Analysis of RIS-Assisted Wireless Communication in Tunnel Scenarios

### 4.1. System Model

**Figure 6:**
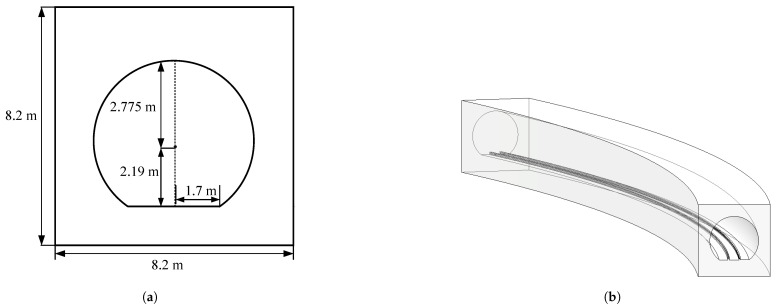
Simulation model of tunnel scenario, they should be listed as: (**a**) Cross-sectional view of the tunnel. (**b**) Three-dimensional model of the tunnel.

To study the performance of RIS-assisted wireless communication for Rx in tunnel scenarios and validate the RIS path loss model proposed in Section 3, we rebuilt a simulation platform using the calibrated model parameters from Section 2. The study focuses on a curved tunnel with a curvature radius of 400 m. The arched cross-section tunnel has a radius of 2.775 m, while the rectangular section has a width of 8.2 m, and the arched bottom section has a width of 3.4 m. The tunnel is made of concrete, with metal tracks at the center. The tunnel cross-section and 3D model are depicted in Figure 6. The tunnel material parameters in the simulation are set according to Table 2.

**Figure 7:**
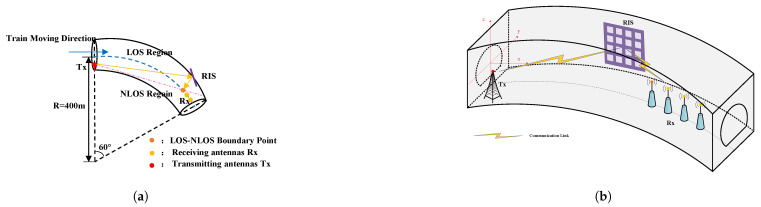
Simulation scheme for RIS-assisted wireless communication in the tunnel scenario, they should be listed as: (**a**) Planar diagram of propagation paths in the tunnel scenario. (**b**) Three-dimensional perspective of propagation paths in the tunnel scenario.

The placement of Tx, Rx, and RIS in the tunnel scenario is illustrated in Figure 7. The Tx is placed on one side of the tunnel wall, while the Rx moves along the central interface of the tunnel, away from the Tx. When a tangent line is drawn from Tx to the tunnel sidewall, the curved tunnel is divided into the LOS region (upper region of the pink line) and the NLOS region (lower region of the pink line). The intersection point of the tangent line and the centerline of the tunnel marks the boundary between the LOS and NLOS regions (represented by the orange dot). The red dot represents the Tx, the yellow dots denote the Rx, the orange dot highlights the LOS-NLOS boundary, and the RIS deployed on the tunnel wall is shown in purple.

When determining the placement of the RIS, the signal propagation characteristics within the tunnel are comprehensively considered, leading to the deployment of the RIS on the tunnel wall. The transmission beam direction of the Tx is set perpendicular to the curvature radius of the tunnel, establishing a geometric relationship where the tunnel center, the Tx, and the RIS form approximately right triangles. Based on this geometric configuration, the specific location of the RIS on the tunnel wall is determined through mathematical calculations employing the sine and cosine laws and the Pythagorean theorem.

In this simulation, the directional Tx is placed on the right side of the tunnel section, with its beam direction perpendicular to the tunnel’s radius of curvature.

**Figure 8:**
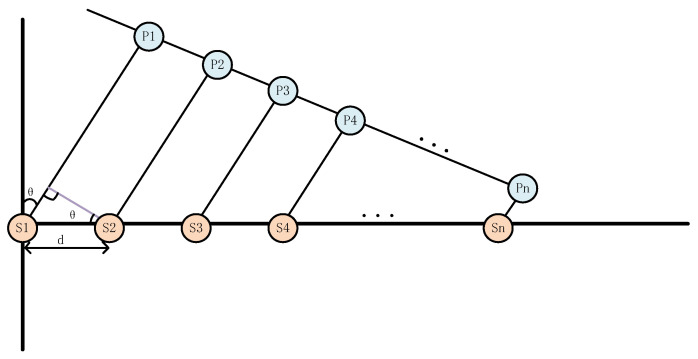
N-element linear array.

Then, we will calculate the half-power beamwidth (HPBW) of the Tx by considering it as an antenna array with a 16 × 16 array. According to the basic theory of electromagnetic field, we first consider the case that the transmit antenna is *N* oscillators arranged in a straight line, as shown in Figure 8 below, each oscillator is the same, so the resulting pattern of the array is the product of the element pattern and the array factor pattern.

We observe that for array antennas, the shape of the radiation pattern is primarily determined by the array factor pattern. Its HPBW can be derived based on the half-power point angle $\theta_{0.5}$ of the main lobe in the array factor pattern. For the side-shooting array, we have:

(15) $$\frac{\sin(N\frac{\pi d}{\lambda }\sin\theta_{0.5})}{N\sin(\frac{\pi d}{\lambda }\sin\theta_{0.5})}=0.707$$

Consequently, we derive:

(16) $$HPBW=2\theta_{0.5}=2\sin^{-1}\left(0.444\frac{\lambda }{Nd}\right)\approx 0.89\frac{\lambda }{Nd}$$

Converting to degrees and denoting L=Nd as the array length, we obtain:

(17) $$HPBW=2\theta_{0.5}=51^{\circ }\frac{\lambda }{Nd}$$

In practical scenarios, the element spacing in the horizontal dimension is typically 0.5λ, while the spacing in the vertical dimension is generally 0.65λ. Thus, when *d* = 0.5λ, *N* = 16, we have HPBW = $6.375^{\circ }$, meaning the HPBW of the antenna’s H-plane is $6.375^{\circ }$. When *d* = 0.65λ, we find HPBW = $4.9^{\circ }$, indicating the HPBW of the antenna’s E-plane is $4.9^{\circ }$.

A single RIS is deployed on the tunnel wall where the beam is acting. The initial receiving point is located 86 m from the Tx, and subsequent Rx are positioned along the tunnel centerline, further away from the Tx. The transmitted signal is a sinusoidal signal with an initial phase of 0, a frequency of 28 GHz, and a power of 20 dBm. The size of the RIS in the simulation is 40 × 40, and the maximum number of reflections is 2. The relevant simulation parameters are set as indicated in Table 3. The specific parameters of the Tx and Rx are presented in Table 4.

### 4.2. Simulation Results and Analysis

**Figure 9:**
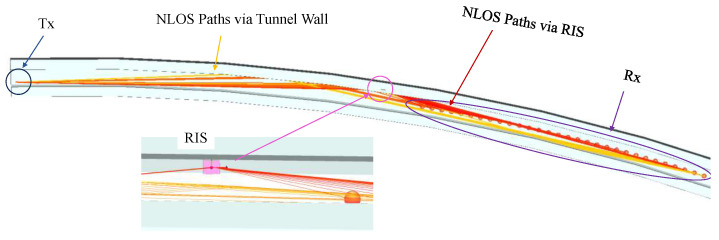
Propagation paths in tunnel scenarios on the WI simulation platform.

Figure 9 illustrates the simulated signal propagation paths within the tunnel. The paths from the Tx to the Rx are primarily composed of NLOS paths via the tunnel wall (orange lines) and NLOS paths reflected by the RIS (red lines). Furthermore, to facilitate observation of the signal propagation, the upper part of the simulated tunnel is set to be available but invisible.

**Figure 10:**
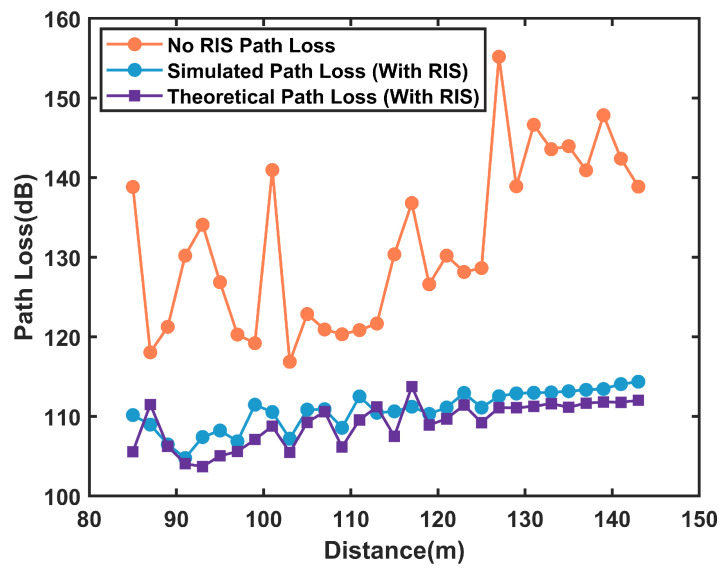
Comparison of Path Loss in Tunnel Scenarios with RIS-Assisted Communication.

Figure 10 compares the path loss with and without RIS in the tunnel scenario, along with the theoretical path loss calculated using Equation (14). The results show that RIS significantly reduces path loss by approximately 20 dB, demonstrating its effectiveness in enhancing signal transmission distance in tunnels. Furthermore, the simulated path loss with RIS closely aligns with the theoretical values, with an MAE of 2.01 dB, which verifies the accuracy of Equation (14).

In order to validate the theoretical model of RIS-based path loss in tunnel scenarios proposed in Section 3 of this article, the differences between the simulated path loss results and the theoretically calculated values were obtained by subtraction. These differences were analyzed using the cumulative distribution function (CDF). It is found that the difference between the simulation results and the theoretical results is step-like and contains negative values. According to the statistical characteristics of the data, we consider using t-distribution and normal distribution fitting. The t-distribution, or more precisely the tLocation-Scale distribution [23,24], can better describe the distribution characteristics of the difference, especially when dealing with fat-tailed characteristics or outliers. The tLocation-Scale distribution can flexibly adjust the shape of the distribution by introducing the location parameter μ, the scale parameter σ, and the degree of freedom parameter ν to more accurately capture the uncertainty in the communication environment. Its probability density function can be expressed as follows.

(18) $$f\left(x\right)=\frac{\Gamma \left(\frac{\nu +1}{2}\right)}{\sigma \sqrt{\nu \pi }\Gamma \left(\frac{\nu }{2}\right)}{\left(1+\frac{{\left(x-\mu \right)}^{2}}{\nu \sigma^{2}}\right)}^{-\frac{\nu +1}{2}}$$

where μ is the location parameter, σ is the scale parameter, ν is the degree of freedom parameter and Γ(·) is the Gamma function.

**Figure 11:**
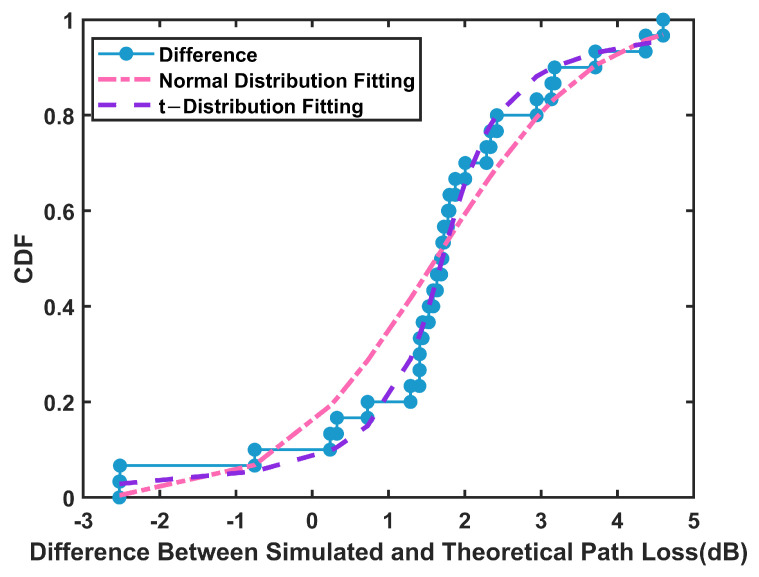
Fitting of simulation and theoretical differences in the tunnel scenario.

The tLocation-Scale distribution with a mean value of 1.7059, a standard deviation of 0.5729, and a degree of freedom of 1.2337 was obtained by fitting, as shown in Figure 11. The results show that this distribution can better reflect the difference in the distribution between simulation and theoretical value.

In summary, the final expression for the RIS-based path loss in the tunnel scenario can be expressed as:

(19) $$PL_{final}=P_{t}-10\mathrm{lg}(\frac{\lambda^{2}\beta }{8\pi \eta_{0}}{\left|\sum_{i=1}^{N_{p}}\left[E_{\theta ,i}\left(\theta_{i},\phi_{i}\right)g_{\theta }\left(\theta_{i},\phi_{i}\right)+E_{\phi ,i}\left(\theta_{i},\phi_{i}\right)g_{\phi }\left(\theta_{i},\phi_{i}\right)\right]\right|}^{2})-30+G_{r}+G_{t}-L_{s}+X$$

where *X* is a random variable with the t-distribution, which in the case depicted in this paper has a mean of 1.7059, a standard deviation of 0.5729, and a degree of freedom of 1.2337, $L_{s}$ is the rest of the loss of the system including the bandwidth overlap factor, *d* is the distance between Tx and Rx, the transmitted power is set to be a known quantity $P_{t}$, $G_{t}$ and $G_{r}$ represent the gains of Tx and Rx.

## 5. Conclusions

This paper uses a systematic simulation of RIS-assisted wireless communication based on the simulation platform built for tunnel scenarios. The RIS path loss model applicable to tunnel environments is derived and verified, and the simulation results prove the accuracy of the formula in describing the path loss. Based on the simulation, to further improve the adaptability and reliability of the model so that it can cover all the signal transmission characteristics in the tunnel scenario, this paper introduces the t-distribution parameter based on the difference between theory and simulation so that the model can more comprehensively reflect the uncertainty in the actual communication environment. The results provide theoretical support for analyzing the channel characteristics of RIS in tunnel scenarios and have guiding significance for designing and optimizing future railway communication systems.

## Figures and Tables

**Table 1:** Simulated antenna parameters.

Parameters	Values
Frequency (GHz)	28
Bandwidth (MHz)	100
Height of transmitting antenna (m)	3.1
Height of receiving antenna (m)	2.7
Antenna type	Omnidirectional antenna
Antenna polarization	Vertical polarization

**Table 2:** Calibrated Simulation Parameters.

Parameters	Values
Roughness (m)	0.04
Thickness (m)	0.5
Conductivity (S/m)	0.4838
Dielectric constant	5.31
Ray spacing (°)	0.1
Number of reflections	6

**Table 3:** Simulation parameter settings.

Parameters	Values
Frequency (GHz)	28
Transmitted power (dBm)	20
RIS array size	40 × 40
dx (m)	0.025
dy (m)	0.025
Transmitting antenna gain (dBi)	20
Receiving antenna gain (dBi)	5
Height of Tx (m)	3.1

**Table 4:** Antenna parameter settings.

	Transmitting Antenna	Receiving Antenna
Antenna type	Directional antenna	Omni-directional antenna
Antenna gain (dBi)	20	5
HPBW	E-plane: 4.9°	/
	H-plane: 6.375°	

## Data Availability

Data are contained within the article.
